# Neo-adjuvant treatment of adenocarcinoma and squamous cell carcinoma of the cervix results in significantly different pathological complete response rates

**DOI:** 10.1186/s12885-018-5007-0

**Published:** 2018-11-12

**Authors:** Karen Couvreur, Eline Naert, Emiel De Jaeghere, Philippe Tummers, Amin Makar, Pieter De Visschere, Mieke Van Bockstal, Jo Van Dorpe, Wilfried De Neve, Hannelore Denys, Katrien Vandecasteele

**Affiliations:** 1 Department of Medical Oncology, UZ Gent, Ghent, Belgium; 2 Department of Gynecology, UZ Gent, Ghent, Belgium; 3 Department of Radiology, UZ Gent, Ghent, Belgium; 4 Department of Pathology, UZ Gent, Ghent, Belgium; 5 Department of Radiation Oncology, UZ Gent, Ghent, Belgium

**Keywords:** Adenocarcinoma, Squamous cell carcinoma, Cervical cancer, Differences, Survival, Prognosis

## Abstract

**Background:**

Previous studies on cervical cancer reported a worse outcome for adenocarcinoma (AC) compared with squamous cell carcinoma (SCC). Nevertheless, standard treatment remains identical. Insight in the impact of histological types on biological behavior and pathological complete response rates might result in a treatment paradigm shift.

**Methods:**

Clinicopathological characteristics, survival rates and relapse patterns were compared between AC (*n* = 36) and SCC (*n* = 143) cervical cancer patients. Pathological response to treatment was evaluated in the patient subgroup treated with neo-adjuvant chemoradiation followed by surgery (NA-CRT group; *n* = 84).

**Results:**

In the entire cohort, 5y Disease Specific Survival (DSS) was 97.1 and 84% for AC and SCC respectively (*p* = 0.150). In the NA-CRT group 5y DSS was 100 and 75.5% for AC and SCC respectively (*p* = 0.059). Relapse patterns did not differ significantly between AC and SCC in the entire cohort, or in the NA-CRT group. Adenocarcinoma patients treated with NA-CRT showed significantly less pathological complete response compared with SCC patients (AC = 7%, SCC = 43%, *p* = 0.027).

**Conclusions:**

There were no statistically significant differences regarding relapse and DSS rates between SCC and AC in the entire cohort, or the NA-CRT group. However, a trend to better 5y DSS of AC in the NA-CRT group was observed. This analysis showed significant differences in treatment responses after NA-CRT: patients with AC responded remarkably less to chemoradiation, resulting in a significantly lower pathological complete response rate. These findings imply a need for a paradigm shift in the treatment of cervical AC patients.

**Electronic supplementary material:**

The online version of this article (10.1186/s12885-018-5007-0) contains supplementary material, which is available to authorized users.

## Background

In 2012, 528.000 new cases of cervical cancer were diagnosed worldwide. With an estimated number of 266.000 cervical cancer deaths in the same year, this accounts for 7.5% of all female cancer deaths globally [1]. About 80% of all cervical cancers are squamous cell carcinomas (SCC), and about 20% are adenocarcinomas (AC). Unusual histological variants are rare and account only for a minority of cases [2].

While some studies reported a worse outcome in AC [3–6], others have failed to observe this difference [7–9]. Several studies reported significantly worse survival in patients with AC (compared to SCC) who received definitive radiation (RT) or chemoradiation (CRT) [10–13]. Others suggest that AC has a worse outcome than SCC when treated with RT compared to RT combined with chemotherapy or surgery [14–17]. These results suggest that AC of the cervix might be less radiosensitive than SCC. Despite etiological, biological and prognostic differences, a specific treatment strategy to tackle AC has not yet been implemented [18]. To date, the recommended management of cervical cancer is mostly independent of its histological subtype but merely guided by staging at diagnosis [18]. For early-stage cervical cancer patients, radical hysterectomy (followed by CRT in case of high-risk for relapse) is the main treatment approach. Only for fertility-sparing surgery (not recommended for patients with small cell neuroendocrine tumors, gastric type adenocarcinoma or adenoma malignum), recommendations differ between AC and SCC. For locally advanced cervical cancer patients, definitive CRT is standard of care [18]. Definitive CRT is a 2-step process consisting of external beam RT ± chemotherapy (if possible cisplatin) and a brachytherapeutic boost. Even with the use of image-guided dose-intensified brachytherapy, local relapse arising from CRT-resistant foci is high (3y-local pelvic control rates of 73% up to 96%, depending on stage and treating center) and remains a major cause of treatment failure [19–21]. In exchange for an improved overall survival (OS), adding chemotherapy to conventional RT has doubled the risk of severe acute hematological and gastro-intestinal toxicity and tripled platelet toxicity [22].

Triggered off by both the high local recurrence and the toxicity rates we challenged the gold standard by investigating the role of surgery after definitive CRT [23–25], allowing a pathological evaluation of treatment response in this specific group of cervical cancer patients.

The goal of this retrospective analysis was to determine the clinicopathological characteristics of patients with cervical cancer treated at a single university center and to investigate the differences in survival and relapse rates between AC and SCC of the cervix. These characteristics, extended with pathological treatment response, have been investigated in the subgroup of patients treated with CRT followed by surgery.

## Methods

### Study population

After institutional ethics committee approval was obtained (B670201628633), the medical records of FIGO stage IA1-IVA cervical cancer patients were reviewed. All patients were treated between 1/1/2005 and 31/12/2015. Twenty-eight out of 207 patients were excluded due to following reasons: treatment for recurrent disease or metastatic disease at diagnosis (*n* = 9 and *n* = 8, respectively), treatment received in another center (*n* = 7), treatment interrupted according to patients’ wish (*n* = 1) or general non-cervical cancer (or its treatment) related problems (*n* = 3). Independent checks were performed for patient, tumor, treatment and outcome characteristics to identify and correct major reporting errors.

The patient cohort was classified according to histological type and FIGO stage: adenocarcinoma (AC) including adenosquamous subtypes (*n* = 36) versus squamous cell carcinoma (SCC; *n* = 143) and early (FIGO stage IA to IB1) versus advanced stages (FIGO stage IB2 to IVA). Following patient data were registered: age, smoking, histological subtype, FIGO stage and TNM classification, tumor grade, presence or absence of lymphovascular space invasion (LVSI) and pelvic lymph node status, thrombocyte count, tumor size and depth of invasion in case of primary surgery (maximum measurable distance in cm), type of treatment (including concomitant administration of chemotherapy), date of diagnosis, date of end of therapy, date of last follow-up, date and localization of *first* relapse, date and cause of death. Thrombocytosis was defined as a platelet count above 450.000/μL.

### Treatment, response evaluation, follow-up and relapse pattern

Pre-treatment imaging consisted of magnetic resonance imaging (MRI) of the pelvis and whole body ^18^Fluoro-deoxyglucose positron emission tomography – computed tomography (^18^FDG PET-CT). The following treatment regimens were used:

1. surgery;
2. surgery + adjuvant chemoradiation (CRT);
3. neo-adjuvant chemoradiation (NA-CRT) + surgery according to the study protocol in case of FIGO stage IB2-IVA [25]. This group contained all patients who were *intended* to undergo surgery, including those with an inoperable tumor due to insufficient response to NA-CRT, and is called the NA-CRT group from this point onwards;
4. definitive CRT (surgery was never intended).

If possible, chemotherapy (C) was administered concomitantly and consisted of weekly single-agent cisplatin (40 mg/m^2^). RT was performed in 25 fractions using an Intensity Modulated Arc Technique up to a) a minimal dose (D98) of 45Gy to the whole target in the adjuvant CRT group and b) a minimal dose (D98) of 45Gy to the elective lymph nodes and target volume and 62/60Gy (using a simultaneously integrated boost) to the tumor/affected lymph nodes in the NA-CRT group (as previously described in detail) [24–26]. From 1/2/2009 onwards, para-aortic lymph node irradiation was performed in case of pathological enlarged (lymph node with shortest axis > 10 mm or round lymph node with axis > 8 mm) or PET-positive pelvic lymph nodes. Brachytherapy was applied a) from stage IB2 onwards in the adjuvant CRT group and b) in case of doubtful or positive margins after surgery in the NA-CRT group.

Surgery consisted of type II Wertheim hysterectomy with pelvic lymphadenectomy performed within 6 to 8 weeks after ending NA-CRT. In the NA-CRT group, selective nodal removal was only performed in case of pathological enlarged or PET-positive pelvic lymph nodes on the diagnostic ^18^FDG PET-CT (instead of pelvic lymphadenectomy). After surgery in case of NA-CRT treatment, response was categorized as: 1) pathological complete response (pCR): no evidence for the presence of viable tumor cells; 2) incomplete pathological response: any amount of viable tumor cells.

Patients were followed-up weekly during treatment, and 1 and 3 months thereafter. Follow-up was scheduled 3-monthly during the first 2 years, 6-monthly during year 3–5 and annually thereafter.

Relapse patterns were categorized into four groups: 1) locoregional relapse (relapse at the tumor site and pelvic lymph nodes); 2) distant nodal relapse (relapse at distant lymph nodes outside the pelvis, including the para-aortic, supraclavicular and inguinal nodes); 3) non-nodal distant relapse (peritoneal, visceral or bone metastases) and; 4) combined (any of previous categories occurring synchronously as 1st event of relapse).

### Survival rate definitions

Overall survival (OS) was defined as the time from date of histological diagnosis to either the date of death due to any cause or the date of last follow-up. In disease-specific survival (DSS) analysis, only deaths caused by cervical cancer or due to a cancer-related cause were considered as events. Disease-free survival (DFS) was defined as the time from end of therapy to the occurrence of relapse. Relapse was confirmed either histologically or clinically when follow-up imaging was highly suggestive for recurrence.

### Statistical analysis

The primary goal was to compare survival rates and relapse pattern between AC and SCC in the entire patient cohort. The secondary goal was to evaluate a difference in survival, relapse pattern and pathological treatment response to NA-CRT between AC and SCC in the NA-CRT group.

Data were analyzed with the R environment for statistical computing [27]. Balanced patient characteristics were tested with nonparametric Wilcoxon tests for equality of means, and Pearson’s chi-square tests for equality of proportions [28, 29]. Kaplan-Meier curves were applied to estimate overall survival (OS), disease-specific survival (DSS) and disease-free survival (DFS) [30]. Univariate and multivariate survival differences between Kaplan-Meier curves were evaluated with the Cox proportional hazards regression and log-rank test [31]. All tests were evaluated with a 95% confidence interval.

## Results

### Patient and treatment characteristics

Data of 179 cervical cancer patients were analyzed, of which 36 were AC and 143 were SCC. Median age at diagnosis was 50 years (range 24–89); median follow-up was 50 months (3–148). FIGO stage distribution was as follows: IA = 15 (8%); IB = 71 (40%); IIA = 6 (3%); IIB = 58 (32%); IIIA = 8 (5%); IIIB = 14 (8%); and IVA = 7 (4%). Age, follow-up period, nodal status, depth of invasion and LVSI were similar between both groups (Table 1). Lymph node metastases were present in 28 and 38% of AC and SCC patients (*p* = 0.319) respectively. In 5 SCC patients nodal status could not be assessed. Mean tumor size was significantly larger for SCC (4.3 cm) than AC (3.5 cm) (*p* = 0.028); ACs were more often stage IB1 (50% versus 25% for SCC; *p* = 0.007) and well differentiated (31% versus 6% in SCC; *p* = 0.0001). Squamous cell carcinomas were more often moderately differentiated than AC (48% versus 25% resp.; *p* = 0.024). The treatment regimens were not significantly different between AC and SCC (Table 2). 14/36 (39%) AC and 70/143 (49%) SCC patients were treated with NA-CRT intent respectively (NA-CRT group). Seven patients (all SCC) did not respond sufficiently (persisting parametrial invasion) to NA-CRT and did not undergo surgery. Apart from median follow-up (AC: 59 m versus SCC: 36 m; *p* = 0.033) and tumor grade (AC were more often well differentiated: 21% versus 3%; *p* = 0.039 or had an unknown differentiation grade: 50% versus 20%; *p* = 0.042) patient characteristics in the NA-CRT group were similar between the AC and SCC groups (Table 1).

**Table 1:** Patient characteristics

*n*	Entire Cohort			Neo-Adjuvant Chemoradiation group		
	AC	SCC	p	AC	SCC	p
	36	143		14	70	
*Age in y; median (range*)	48 (27–82)	51 (24–89)	0.196	47.5 (38–65)	57 (24–89)	0.070
*Follow-up in m; median (range)*	54 (7–138)	50 (3–148)	0.218	59 (7–135)	36 (7–133)	**0.033**
*Tumor FIGO stage, n (%)*						
IA	1 (3)	1 (1)	0.862	0	0	
IA1	0 (0)	5 (3)	0.567	0	0	
IA2	0 (0)	8 (6)	0.317	0	0	
IB	2 (6)	2 (1)	0.380	0	0	
IB1	18 (50)	36 (25)	**0.007**	1 (7)	1 (1)	0.749
IB2	2 (6)	11 (8)	0.934	2 (14)	6 (9)	0.868
IIA	0 (0)	2 (1)	1	0	0	
IIA1	0 (0)	1 (1)	1	0	0	
IIA2	2 (6)	1 (1)	0.193	1 (7)	0	0.368
IIB	9 (25)	49 (34)	0.388	8 (57)	43 (61)	1
IIIA	0 (0)	8 (6)	0.317	0	6 (9)	0.570
IIIB	2 (6)	12 (8)	0.826	2 (14)	8 (11)	1
IVA	0 (0)	7 (5)	0.382	0	6 (9)	0.570
*Tumor TNM stage, n (%)*						
I	21 (58)	52 (36)	**0.027**	2 (14)	2 (3)	0.251
II	8 (22)	33 (23)	1	7 (50)	27 (39)	0.619
III	7 (19)	50 (35)	0.113	5 (36)	34 (49)	0.557
IV	0 (0)	8 (6)	0.317	0 (0)	7 (10)	0.480
*Pelvic lymph node status, n (%)*						
negative	26 (72)	83 (58)	0.172	11 (79)	34 (49)	0.078
positive	10 (28)	55 (38)	0.319	3 (21)	36 (51)	0.078
unknown, n (%)	0 (0)	5 (3)	0.567	0 (0)	0 (0)	1
*Tumor size, cm*						
median (range)	3.5 (0.4–7.0)	4.3 (0.1–10.5)	**0.028**	4.3 (2.3–7)	5.5 (1.8–8.2)	0.056
unknown, n (%)	2 (6)	24 (17)	0.149	0	2 (3)	1
*Depth invasion, cm*						
median (range)	1 (0.3–1.7)	0.8 (0.1–5)	0.905	–	–	
primary surgery, n (%)	13 (36)	46 (32)	0.801	–	–	
unknown, n (%)	9 (25)	14 (10)	**0.031**	–	–	
no primary surgery, n (%)	14 (39)	83 (58)	0.061	–	–	
*Chemotherapy concomitant, n (%)*						
*yes*	–	–		13 (93)	62 (89)	1
*no*	–	–		1 (7)	8 (11)	1
*Differentiation status, n (%)*						
well	11 (31)	9 (6)	**0.0001**	3 (21)	2 (3)	**0.039**
moderate	9 (25)	68 (48)	**0.024**	3 (21)	32 (46)	0.166
poor	7 (19)	43 (30)	0.288	1 (8)	22 (32)	0.126
unknown	9 (25)	23 (16)	0.315	7 (50)	14 (20)	**0.043**
*LVSI, n(%)*						
negative	7 (19)	14 (10)	0.187	–	–	
positive	11 (31)	32 (22)	0.419	–	–	
unknown	4 (11)	14 (10)	1	–	–	
no primary surgery	14 (39)	83 (58)	0.061	–	–	
*Patient outcome, n (%)*						
alive/censored	33 (92)	107 (75)	**0.0498**	13 (93)	46 (66)	0.088
disease-specific death	2 (6)	20 (14)	0.274	0	15 (21)	0.126
other cause of death	0 (0)	14 (10)	0.107	0	8 (11)	0.406
unknown	1 (3)	2 (1)	1	1 (7)	1 (1)	0.749

*AC* adenocarcinoma, *SCC* squamous cell carcinoma, *n* amount, *p* p-value, *y* years, *LVSI* lymph vascular space invasion, significant p-values (*p* < 0.05) are presented in bold

**Table 2:** Primary treatment by tumor histology

Treatment, n (%)	AC	SCC	p
Surgery	10 (28)	23 (16)	0.169
Surgery + adjuvant chemoradiation^ab^	12 (33)	36 (25)	0.437
Neo-adjuvant chemoradiation^a^ with intent for surgery^c^	14 (39)	70 (49)	0.371
Definitive chemoradiation^ad^	0 (0)	12 (8)	0.154
Brachytherapy alone^e^	0 (0)	1 (1)	1
Neo-adjuvant chemotherapy + conization^f^	0 (0)	1 (1)	1

*AC* adenocarcinoma (n = 36), *SCC* Squamous Cell Carcinoma (n = 143); ^a^ If possible, chemotherapy (C) was administered concomitantly and consisted of weekly single-agent cisplatin at 40 mg/m^2^; ^b^ In 11/33 AC/SCC patients this includes a brachytherapeutic boost; ^c^ 7 SCC patients did not receive surgery and 1/3 AC/SCC patients received a brachytherapeutic boost due to positive or doubtful margins; ^e^ due to co-morbidities; ^f^ fertility sparing

### Outcome

#### Survival

**Fig. 1:**
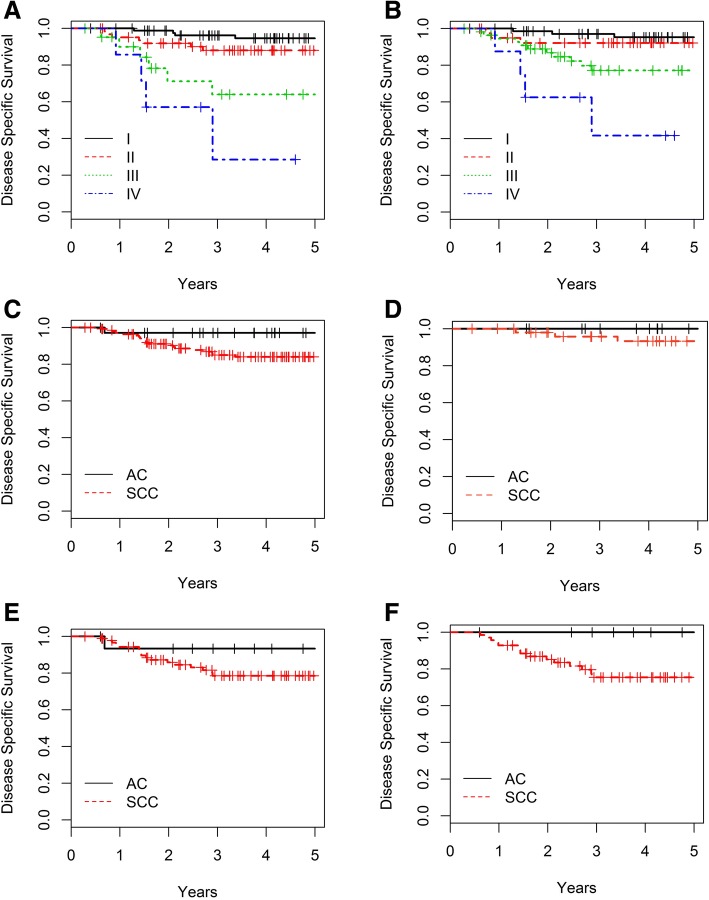
**a**. Disease-Specific Survival (DSS) and FIGO stage: I (*n* = 86, 5y-DSS 94.7%), II (*n* = 64, 5y-DSS 88.0%), III (*n* = 22, 5y-DSS 64.1%) and IV (*n* = 7, 5y-DSS 28.6%). (III and IV, *p* < 0.001). **b**. DSS and TNM stage: I (*n* = 73, 5y-DSS 95.3%), II (*n* = 41, 5y-DSS 92.2%), III (*n* = 57, 5y-DSS 77.2%) and IV (*n* = 8, 5y-DSS 41.7%). (III and IV, p < 0.001). C-F. DSS and histology: **c**. Entire cohort: adenocarcinoma (AC): *n* = 36, 5y- DSS: 97.1% and Squamous Cell Carcinoma (SCC): *n* = 143, 5y-DSS 84.0%; *p* = 0.150. **d**. Entire cohort; early stages: AC: *n* = 20, 5y-DSS: 100% and SCC: *n* = 52, 5y-DSS: 93.3%; *p* = 0.847. **e**. Entire cohort; advanced stages: AC: *n* = 16, 5y-DSS: 93.3% and SCC: *n* = 91, 5y-DSS: 78.6%; *p* = 0.232. **f**. NA-CRT group: AC: n = 14, 5y-DSS: 100% and SCC: *n* = 70; 5y-DSS 75.5%; *p* = 0.059

Thirty-nine deaths were reported, 3 in the AC group and 36 in the SCC, resulting in a 5y OS rate of 94.4 and 73.2% (*p* = 0.034) respectively. The 5y OS rate for early (FIGO stage IA to IB1) AC and SCC was 100 and 89.4% (*p* = 0.408) respectively. The 5y OS for advanced (FIGO stage IB2 to IVA) AC and SCC was 87.5 and 63.1% respectively (*p* = 0.120). The SCC group showed more non-disease-specific deaths (14/36, 39% of all SCC deaths) compared to the AC group (0/3) (Table 1). Although this difference was not statistically significant (*p* = 0.107), we preferred using DSS to correct for this random effect. Both FIGO (5-y DSS is 94.7, 88, 64.1 and 28.6% in FIGO stage I, II, III and IV respectively) and TNM stage (5-y DSS is 95.3, 92.2, 77.2 and 41.7% in TNM stage I, II, III and IV respectively) had an impact on 5y DSS (Fig. 1a and b). The estimated 5y DSS in the entire cohort, AC and SCC group amounted 86.8, 97.1 and 84.0% respectively (Fig. 1c), (*p* = 0.150). The estimated 5y DSS did not differ significantly between AC and SCC in both the early and advanced stage cohort (AC/SCC: early 100%/93.3% and advanced 93.3%/78.6%; *p* = 0.847/0.232) (Fig. 1d and e).

Five-year DSS amounted 79.8% in the overall NA-CRT group (*n* = 84), and it was 100 and 75.5% (*p* = 0.060) for the AC (*n* = 14) and SCC patients (*n* = 70) in this group respectively (Fig. 1f).

**Fig. 2:**
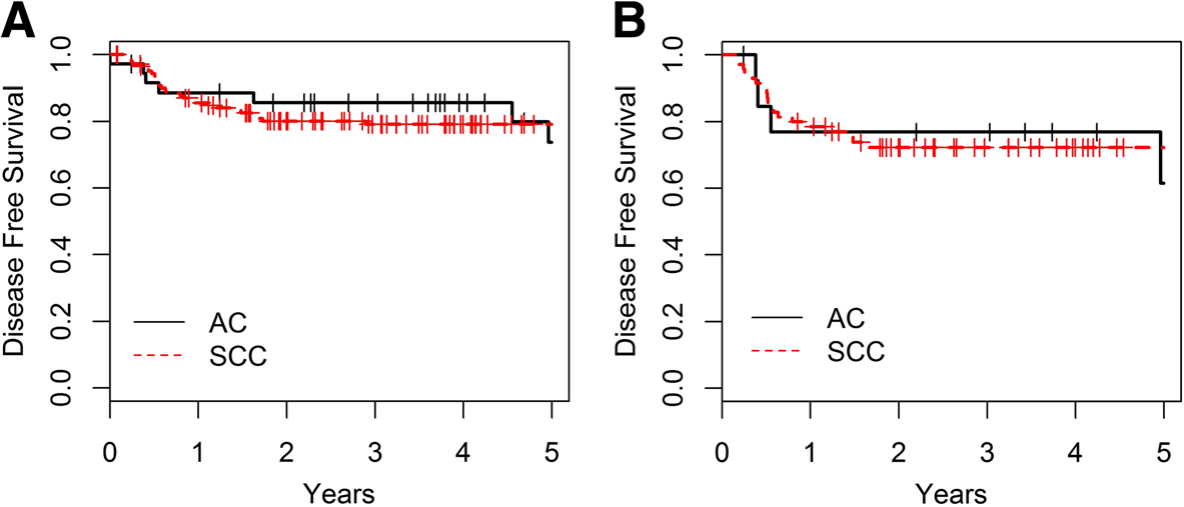
Disease-Free Survival and histology: **a**. Entire cohort. Adenocarcinoma (AC): *n* = 36; 5y-DFS 73.8% and Squamous Cell Carcinoma (SCC): *n* = 143, 5y-DFS 79.2%; *p* = 0.809. **b**. NA-CRT group. AC: n = 14; 5y-DFS 61.5% and SCC: n = 70, 5y-DFS 72.3%; *p* = 0.558. c

The estimated 5-year DFS rates were 73.8 and 79.2% (*p* = 0.809) in the entire cohort for the AC and SCC group respectively. The estimated 5-year DFS rates were 61.5 and 72.3% (*p* = 0.558) in the NA-CRT group for AC and SCC respectively (Fig**.** 2a and b).

#### Pathological complete response rates in the NA-CRT group

Seventy-seven patients treated with NA-CRT intent were operated upon. A pCR was obtained in 7% (1/14) and 43% (27/63) of the AC and SCC patients respectively (*p* = 0.027). This difference remained statistically significant when all non-operated tumors (n = 7, all SCC) were considered as incomplete pathological response: 7% (1/14) versus 39% (27/70) pCR for AC and SCC respectively (*p* = 0.049).

#### Relapse

The relapse rate for the entire cohort was 8/36 (22%) and 28/143 (20%) for AC and SCC respectively (*p* = 0.904) (Additional file 1: Table S1). In AC, site of first relapse was 1/8 (12.5%) locoregional, 1/8 (12.5%) distant nodal, 4/8 (50%) distant non-nodal and 2/8 (25%) a combination of previous categories. In SCC, the site of first relapse was 11/28 (39%) locoregional, 1/28 (4%) distant nodal, 5/28 (18%) distant non-nodal and in 11/28 (39%) of the cases a combination of previous categories. No significant difference was found. In the NA-CRT group, relapse rate and pattern were not significantly different: 5/14 (36%) and 19/70 (27%) for AC and SCC respectively (*p* = 0.746). In AC, the site of first relapse was 0/5 (0%) locoregional, 1/5 (20%) distant nodal, 3/5 (60%) distant non-nodal and in 1/5 (20%) of cases a combination of previous categories. In SCC, the site of first relapse was 8/19 (42%) locoregional, 1/19 (5%) distant nodal, 3/19 (16%) distant non-nodal and 7/19 (37%) combined.

### Uni- and multivariate analysis

Univariate analysis (Additional file 1: Table S2) showed that lymph node involvement (HR = 2.968, *p* = 0.016), tumor size (HR/cm = 1.53) and advanced FIGO stage (HR = 3.58, *p* = 0.021) significantly influenced DSS. Thrombocytosis, tumor differentiation and LVSI did not. In multivariate analysis, only FIGO stage was observed to have a significant impact on DSS (Additional file 1: Table S3).

## Discussion

In clinical practice, AC seems to be less responsive to therapy and more frequently associated with earlier distant metastases. Population-based studies indicate a rising incidence of AC despite cytological screening [32]. In addition, recent studies identified certain subtypes of non-HPV related adenocarcinoma, like gastric subtypes with worse prognosis and higher tendency for adnexal and distant metastases. Although a more aggressive approach of AC has often been subject of intensive research, treatment of both histological types of cervical cancer currently remains almost identical. The purpose of this study was to test the hypothesis that both histological subtypes of cervical carcinoma have different survival, relapse patterns and response rates to treatment and thus require tailored therapy.

There are several limitations to this study, some of them inherent to the retrospective design, such as recall and confounding bias. LVSI and depth invasion were often missing in the histopathological reports. In addition, our study reports on a heterogeneous group of patients including all stages with different types of treatment. Analyses were not corrected for treatment type. All consecutively treated patients within the reported period were included. Therefore, all findings are restricted to this population sample. A power analysis was conducted, and the significance tests had 80% power to detect a difference in DSS of 12% or more between advanced AC and SCC patients. A larger sample size is needed to detect smaller survival differences. Nevertheless, despite the aforementioned limitations, we observed some very interesting and intriguing phenomena.

The study population was well balanced with the exception of FIGO stage IB1 and TNM stage 1 patients, smaller tumors and well differentiated tumors that were overrepresented in the AC population. Moderately differentiated tumors were overrepresented in the SCC population. A higher percentage of stage I [13] and well differentiated [33] AC patients was also observed by others. Despite the overrepresentation of FIGO stage IB1, small and well differentiated tumors in the AC population, we failed to observe a difference in treatment regimen between AC and SCC. We assume that this lack of difference is due to the small patient sample size and the high amount of treatment options. If we reduce the treatment options to primary surgical (including adjuvant CRT or fertility sparing neo-adjuvant chemotherapy and conization) and primary RT intent (including NA-CRT; definitive CRT and brachytherapy alone) we do find more AC (22/36) than SCC (60/143) patients primarily operated upon (*p* = 0.039).

In contrast to our study, several studies did report a worse prognosis for AC (Table 3) [3, 4, 6–8, 34–38]. Three out of 8 early AC/SCC and 9 out of 19 advanced AC/SCC comparisons show a significant difference in survival, all resulting in a worse outcome for AC compared to SCC. Most of the statistically non-significant comparisons had limited AC population sizes, limiting the power of the respective tests. In addition, if the variations in the results reflect normal study-to-study differences, then we also would expect to find studies claiming better AC prognosis. Yet to the best of our knowledge, we failed to find any study reporting a significant better AC survival. In this report, 5y OS of advanced stage AC is 87.5%, which is higher than the results of most studies reported in Table 3 (5 to 87%) [39]. No significant difference in DSS was noted between AC and SCC in the entire study cohort. However, a trend towards a better DSS for AC was seen in the NA-CRT group (*p* = 0.059). A possible explanation is the more aggressive treatment used, where 78% of the advanced stage patients had surgery after NA-CRT. The randomized trial of Landoni et al. compared surgery to RT in patients with FIGO IB-IIA CC and reported equivalent survival rates [15]. However, patients with AC treated with hysterectomy had better DFS than patients treated with definitive RT (66% vs 47%, *p* = 0.02) suggesting that AC may be more resistant to RT [15]. Other reports confirmed that AC of the cervix might be less radiosensitive than SCC [10–13, 40]. In a group of patients treated with definitive CRT, Chen et al. [10] showed that a complete pathological treatment response was significantly more present in the SCC subtype compared to the adenosquamous/AC subtypes (87.1% v s 71.4%, *p* = 0.018), and nearly 1/3 of the adenosquamous/AC subtypes had persistent tumor at the cervix 3 months after completing RT. Huang et al. [11] reported 41% residual disease in the cervix after definitive RT for AC. Our results complement these findings in advanced stage cervical cancer, where pCR is significantly less present in AC (AC = 7%, SCC = 43%, *p* = 0.027). This is not due to FIGO stage or size of the tumor since the AC group had more favorable patient characteristics (smaller tumor size, well differentiated, Table 1). Several potential mechanisms of radioresistance and predictors of treatment response of AC have been described before. Cyclooxygenase-2 (COX-2) was found to be more present in AC and COX-2 negative disease was found in all patients responding to RT [41]. In addition, the anti-apoptotic protein Villin1 was expressed only in cervical AC and its presence is strongly correlated with poorer survival [42]. Others suggested a role for the b-catenin pathway [43]. Exploration of these possible biomarkers of treatment response will be subject of future research.

**Table 3:** Literature Overview

Source	Period	Stage	AC		SCC		Sign.	AC - SCC
			n	5y OS	n	5yOS		
Early								
Hopkins et al. [4]	1970–1985	I	124	0.6	370	0.9	X	−0.3
Shingleton et al. [30]	1984–1990	I	174	0.84	1136	0.84		0
Couvreur et al.^a^	2005–2015	IA-1B1	20	1	52	0.89		0.11
Teke et al. [8]	1996–2000	IA-IIB	33	0.77	218	0.73		0.04
Ayhan et al. [31]	1980–1997	IB	67	0.84	454	0.88		− 0.04
Eifel et al. [32]	1960–1989	IB	229	0.72	1538	0.81	X	− 0.09
Nakanishi et al. [34]	1976–1995	IB	104	0.88	405	0.96	X	−0.08
Galic et al. [3]	1998–2005	IB1	1094	0.85	3214	0.88		−0.03
Shimada et al. [6]	1997–2003	IB1	184	0.92	258	0.95		−0.03
Advanced								
Galic et al. [3]	1998–2005	IB2	343	0.68	1701	0.69		−0.01
Shimada et al. [6]	1997–2003	IB2	39	0.76	67	0.74		0.01
Irie et al. [33]	1981–1996	IB-IIA	50	0.78	198	0.92		−0.14
Shimada et al. [6]	1997–2003	IB-IIB	280	0.87	540	0.83		0.04
Hopkins et al. [4]	1970–1985	II	40	0.47	186	0.62	X	−0.15
Shingleton et al. [30]	1984–1990	II	102	0.57	1073	0.67	X	−0.11
Galic et al. [3]	1998–2005	IIA	202	0.46	1488	0.58	X	−0.13
Shimada et al. [6]	1997–2003	IIA	11	0.55	83	0.87	X	−0.33
Galic et al. [3]	1998–2005	IIB	424	0.46	3754	0.55	X	−0.09
Katanyoo et al. [7]	1980–1997	IIB	85	0.72	170	0.71		0.01
Shimada et al. [6]	1997–2003	IIB	46	0.63	132	0.79	X	−0.16
Couvreur et al.^a^	2005–2015	IB2-IVA	16	0.88	91	0.63		0.25
Hopkins et al. [4]	1970–1985	III	25	0.08	114	0.36	X	−0.28
Shingleton et al. [30]	1984–1990	III	47	0.3	672	0.4		−0.1
Galic et al. [3]	1998–2005	IIIA	80	0.16	695	0.34	X	−0.18
Galic et al. [3]	1998–2005	IIIB	238	0.2	2568	0.31	X	−0.11
Katanyoo et al. [7]	1995–2008	IIIB-IVA	56	0.41	112	0.47		−0.06
Shingleton et al. [30]	1984–1990	IV	41	0.05	287	0.13		−0.08
Galic et al. [3]	1998–2005	IVA	82	0.08	622	0.17		−0.09
Galic et al. [3]	1998–2005	IVB	281	0.09	959	0.06		0.04

*AC* adenocarcinoma, *SCC* Squamous cell carcinoma, *n* number, *5yOS* 5 year overall survival period: years of enrollment of patients, *Sign* significance with p-value < 0.05 (all significance levels lie between 0.01 and 0.05); AC-SCC: 5y survival data AC minus 5y survival data SCC resulting in a delta value

^a^current article

Although pCR was low for AC in the NA-CRT group, locoregional control was 100% and 5y DSS was high: 100% (compared to 75.5% for SCC; *p* = 0.059). This emphasizes the possible role of hysterectomy after NA-CRT, already suggested by Eifel et al. in 1995 (albeit in the era before concurrent chemotherapy) [44].

## Conclusion

To conclude, no statistically significant differences in relapse (incidence and pattern) and DSS between SCC and AC were found. However, a trend towards a better 5y DSS of AC in the NA-CRT group is noticed, despite a significantly lower response to treatment (pCR). The lower pCR rate in the AC subgroup suggests that AC is less radiosensitive than SCC and requires a different therapeutic strategy instead of definitive chemoradiation alone.

## Additional files

Additional file 1: **Table S1.** Relapse pattern in the entire cohort and neo-adjuvant chemoradiation group. **Table S2.** Univariate analysis of DSS. **Table S3.** Multivariate analysis of DSS. (DOCX 26 kb)

## Abbreviations

AC: Adenocarcinoma
C: Chemotherapy
COX-2: Cyclooxygenase-2
CRT: Chemoradiation
DFS: Disease Free Survival
DSS: Disease Specific Survival
FIGO: Fédération Internationale de Gynécologie et d’Obstétrique
Gy: Gray
LVSI: Lymph Vascular Space Invasion
MRI: Magnetic Resonance Imaging
NA-CRT: Neo-adjuvant chemoradiation
OS: Overall Survival
pCR: Pathologic Complete Response
RT: Radiation
SCC: Squamous Cell Carcinoma
^18^FDG PET-CT: ^18^Fluoro-deoxyglucose positron emission tomography
