# Supplementary material for: Neo-adjuvant treatment of adenocarcinoma and squamous cell carcinoma of the cervix results in significantly different pathological complete response rates
Source: BMC Cancer. 2018 Nov 12;18:1101. doi: 10.1186/s12885-018-5007-0 (PMC6233604; doi:10.1186/s12885-018-5007-0)
Supplement: Supplementary file 1 — Table S1. Relapse pattern in the entire cohort and neo-adjuvant chemoradiation group. Table S2. Univariate analysis of DSS. Table S3. Multivariate analysis of DSS. (DOCX 26 kb) [file 12885_2018_5007_MOESM1_ESM.docx]

SUPPLEMENTAL TABLES Table S1: Relapse pattern

| \|  \|  \| **All patients** \| \| \|  \| **NA-CRT Intent patients** \| \| \| \| --- \| --- \| --- \| --- \| --- \| --- \| --- \| --- \| --- \| \|  \|  \| **AC** \| **SCC** \| **p-value** \|  \| **AC** \| **SCC** \| **p-value** \| \| n \| \| 36 \| 143 \|  \|  \| 14 \| 70 \|  \| \| Relapse rate, n (%) \| \| 8 (22.2) \| 28 (19.6) \| 0.9 \|  \| 5 (35.7) \| 19 (27.1) \| 0.75 \| \|  \| Locoregional \| 1(12.5) \| 11(39) \| 0.5 \|  \| 0 (0) \| 8 (42) \| 0.21 \| \|  \| Distant nodal \| 1(12.5) \| 1(4) \| 0.86 \|  \| 1 (20) \| 1 (5) \| 0.88 \| \|  \| Non-nodal \| 4(50) \| 5(18) \| 0.15 \|  \| 3 (60) \| 3 (16) \| 0.15 \| \|  \| Combined \| 2(25) \| 11(39) \| 0.93 \|  \| 1 (20) \| 7 (37) \| 0.86 \|   *AC: adenocarcinoma; SCC: Squamous Cell Carcinoma; NA-CRT: neo-adjuvant chemoradiation.* |
| --- | --- | --- | --- | --- | --- | --- | --- | --- | --- | --- | --- | --- | --- | --- | --- | --- | --- | --- | --- | --- | --- | --- | --- | --- | --- | --- | --- | --- | --- | --- | --- | --- | --- | --- | --- | --- | --- | --- | --- | --- | --- | --- | --- | --- | --- | --- | --- | --- | --- | --- | --- | --- | --- | --- | --- | --- | --- | --- | --- | --- | --- | --- | --- | --- | --- | --- | --- | --- | --- | --- | --- | --- |

### Table S2: Univariate analysis of DSS

| **Parameter** | | **HR** | **95% CI** | **p-value** |
| --- | --- | --- | --- | --- |
| Age | |  |  |  |
|  | HR/year | 1.02 | (0.99-1.05) | 0.21 |
| Tumor FIGO stage | |  |  |  |
|  | I | 1 |  |  |
|  | II | 2.12 | (0.67- 6.69) | 0.20 |
|  | III | 7.115 | (2.16- 23.41) | 0.0012 (**) |
|  | IV | 15.94 | (4.19 - 60.62) | <0.001 (***) |
| Cell type | |  |  |  |
|  | AC | 1 |  |  |
|  | SCC | 0.36 | (0.65-11.9) | 0.15 |
| Thrombocytosis | |  |  |  |
|  | Normal | 1 |  |  |
|  | High | 3.22 | (0.73-14.23) | 0.1 |
| Smoking | |  |  |  |
|  | Non-smoking | 1 |  |  |
|  | Smoking | 0.64 | (0.24-1.68) | 0.29 |
| Tumor size (max) | |  |  |  |
|  | HR/cm | 1.53 | (1.22-1.91) | <0.001 (***) |
| Tumor differentiation | |  |  |  |
|  | well | 1 |  |  |
|  | moderate | 2.08 | (0.25-16.94) | 0.49 |
|  | poor | 4.02 | (0.51-31.8) | 0.19 |
| Lympho-vascular space invasion | | |  |  |
|  | Negative | 1 |  |  |
|  | Positive | 0.53 | (0.07-3.78) | 0.53 |
| Lymph node status | |  |  |  |
|  | Negative | 1 |  |  |
|  | Positive | 2.97 | (1.23-7.18) | 0.01 (*) |

### *HR: Hazard Risk; CI: Confidence Interval; AC: adenocarcinoma; SCC: Squamous Cell Carcinoma; cm: centimeter; max: maximal diameter. Signif. codes: 0 '***' 0.001 '**' 0.01 '*' 0.05 '*

### Table S3: Multivariate analysis of DSS

|  | **Parameter** | **HR** | **95% CI** | **p-value** |
| --- | --- | --- | --- | --- |
| Nodal status | |  |  |  |
|  | Negative | 1 |  |  |
|  | Positive | 1.89 | [0.72-4.98] | 0.20 |
| Histology | |  |  |  |
|  | AC | 1 |  |  |
|  | SCC | 1.59 | [0.35-7.12] | 0.55 |
| Tumor FIGO stage | |  |  |  |
|  | I | 1 |  |  |
|  | II | 2.160 | [0.62-7.48] | 0.22 |
|  | III | 7.160 | [1.98-25.93] | 0.003(**) |
|  | IV | 10.940 | [2.38-50.38] | 0.002(**) |

*HR: Hazard Risk; CI: confidence interval; AC: adenocarcinoma; SCC: Squamous Cell Carcinoma; Significance codes: 0 '***' 0.001 '**' 0.01 '*' 0.05 '*
